# Clinical testing of transcriptome-wide expression profiles in high-risk localized and metastatic prostate cancer starting androgen deprivation therapy: an ancillary study of the STAMPEDE abiraterone Phase 3 trial

**DOI:** 10.21203/rs.3.rs-2488586/v1

**Published:** 2023-02-08

**Authors:** Marina A. Parry, Emily Grist, Larissa Mendes, Peter Dutey-Magni, Ashwin Sachdeva, Christopher Brawley, Laura Murphy, James Proudfoot, Sharanpreet Lall, Yang Liu, Stefanie Friedrich, Mazlina Ismail, Alex Hoyle, Adnan Ali, Aine Haran, Anna Wingate, Leila Zakka, Daniel Wetterskog, Claire L. Amos, Nafisah B. Atako, Victoria Wang, Hannah L. Rush, Robert J. Jones, Hing Leung, William R. Cross, Silke Gillessen, Chris C. Parker, Simon Chowdhury, STAMPEDE collaborators, Tamara Lotan, Teresa Marafioti, Alfonso Urbanucci, Edward M. Schaeffer, Daniel E. Spratt, David Waugh, Thomas Powles, Daniel M. Berney, Matthew R. Sydes, Mahesh K.B. Parmar, Anis A. Hamid, Felix Y. Feng, Christopher J. Sweeney, Elai Davicioni, Noel W. Clarke, Nicholas D. James, Louise C. Brown, Gerhardt Attard

**Affiliations:** 1 Cancer Institute, University College London; London, UK.; 2 MRC Clinical Trials Unit at University College London, Institute of Clinical Trials and Methodology, University College London; London, UK.; 3 Genito-Urinary Cancer Research Group, Division of Cancer Sciences, Manchester Cancer Research Centre, The University of Manchester; Manchester, UK.; 4 Veracyte, Inc; San Diego, USA.; 5 Department of Data Science, Dana-Farber Cancer Institute; Boston, USA.; 6 University of Glasgow, Beatson West of Scotland Cancer Centre; Glasgow, UK.; 7 St James’s University Hospital; Leeds, UK.; 8 Istituto Oncologico della Svizzera Italiana, EOC; Bellinzona, Switzerland.; 9 Università della Svizzera Italiana; Lugano, Switzerland.; 10 Royal Marsden NHS Foundation Trust and Institute of Cancer Research; London, UK.; 11 Guy’s and St Thomas’ NHS Foundation Trust; London, UK.; 12 Johns Hopkins University School of Medicine; Baltimore, USA.; 13 Department of Tumor Biology, Institute for Cancer Research, Oslo University Hospital; Oslo, Norway.; 14 Prostate Cancer Research Center, Faculty of Medicine and Health Technology, Tampere University and Tays Cancer Center, Tampere University Hospital; Tampere, Finland.; 15 Department of Urology, Northwestern University Feinberg School of Medicine; Chicago, USA.; 16 Department of Radiation Oncology, University Hospitals Seidman Cancer Center, Case Comprehensive Cancer Center; Cleveland, USA.; 17 Queensland University of Technology; Brisbane, Australia.; 18 Barts Experimental Cancer Medicine Centre, Barts Cancer Institute, Queen Mary University of London; London, UK.; 19 Barts Cancer Institute, Queen Mary University of London; London, UK.; 20 Department of Medical Oncology, Dana-Farber Cancer Institute; Boston, USA.; 21 University of California San Francisco; San Francisco, USA.; 22 Department of Surgery, The Christie and Salford Royal Hospitals; Manchester, UK.

**Keywords:** Prostate cancer, high-risk localized, synchronous metastatic, transcriptome-wide profiling, mRNA, prognosis, Decipher score, interferon signalling, STAMPEDE trial

## Abstract

Metastatic and high-risk localized prostate cancer respond to hormone therapy but outcomes vary. Following a pre-specified statistical plan, we used Cox models adjusted for clinical variables to test associations with survival of multi-gene expression-based classifiers from 781 patients randomized to androgen deprivation with or without abiraterone in the STAMPEDE trial. Decipher score was strongly prognostic (p<2×10^−5^) and identified clinically-relevant differences in absolute benefit, especially for localized cancers. In metastatic disease, classifiers of proliferation, PTEN or TP53 loss and treatment-persistent cells were prognostic. In localized disease, androgen receptor activity was protective whilst interferon signaling (that strongly associated with tumor lymphocyte infiltration) was detrimental. Post-Operative Radiation-Therapy Outcomes Score was prognostic in localized but not metastatic disease (interaction p=0.0001) suggesting the impact of tumor biology on clinical outcome is context-dependent on metastatic state. Transcriptome-wide testing has clinical utility for advanced prostate cancer and identified worse outcomes for localized cancers with tumor-promoting inflammation.

## INTRODUCTION

Intensification of hormone therapy improves survival and is now standard care for patients with high-risk localized or metastatic prostate cancer starting long-term androgen deprivation therapy (ADT). Whilst disease burden at diagnosis is prognostic, the outcomes for patients balanced by clinical features are highly variable: some patients die from their disease within months and others remain in remission for many years. Transcriptome classifiers, originally studied in breast cancer^1–3^, have shown potential utiity for low- and intermediate-risk localized prostate cancer^4–8^ but have not been clinically implemented in advanced prostate cancer. This could be a missed opportunity. To address this, we used a clinical-grade test to generate transcriptome-wide expression profiles on diagnostic tissue from men randomized to ADT or ADT combined with abiraterone acetate and prednisolone (hereafter referred to as abiraterone) in a multi-centre phase 3 trial conducted within the STAMPEDE platform protocol^9^. This trial reported that addition of abiraterone to ADT improved survival when given continuously to progression for patients with metastatic disease and for two years with prostate radiotherapy to high-risk localized disease patients^9,10^. Other trials have reported a similar benefit for patients with metastatic disease with abiraterone or other second-generation hormone therapies^11–16^. The STAMPEDE trial prospectively stratified patients by the presence or absence of distant metastases (metastatic state) on conventional imaging. Patients with no distant metastases (M0, classified high-risk localized) were stratified by disease burden, namely whether they had prostate only (M0N0) or pelvic nodal spread (M0N1). Patients with metastatic disease (M1) were further sub-divided by disease burden into high and low volume based on the classification used in the CHAARTED trial^17,18^. To test the clinical utility of transcriptome-wide analysis in advanced prostate cancer, we then derived expression-based, multi-gene signature scores and linked these with detailed long-term, protocol-defined follow-up.

## RESULTS

### Cohort summary

**Figure 1:**
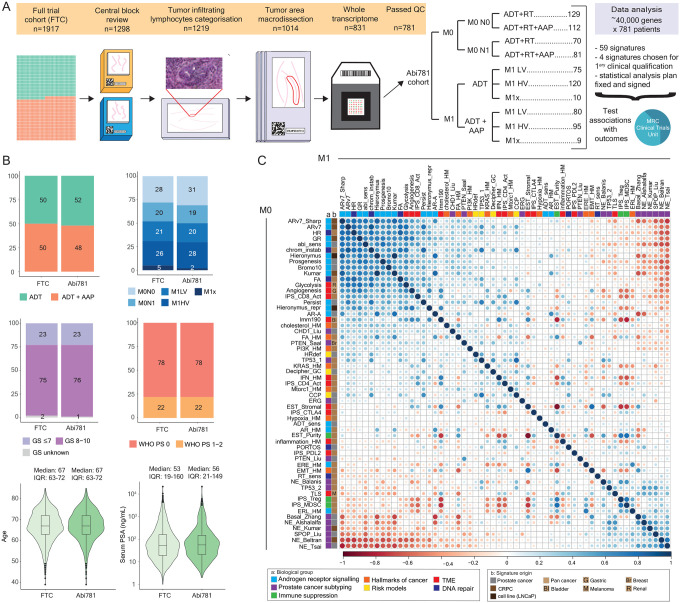
Cohort and signature overview. **A**. Experimental and analytical workflow showing total eligible patients in full trial cohort (FTC) and progress to our Abi781 cohort (additional details in Supplemental Figure 1) with breakdown of Abi781 by treatment arm and disease burden. Patients with high-risk localized (M0) disease were stratified by nodal status at randomization and patients with metastatic (M1) disease were assigned burden status following post-hoc central review, where scans were available (M1x indicates cases where burden status could not be assigned). **B**. Stacked barplots and box-violin plots comparing proportion or range of baseline clinical features that were adjusted for in prognostic analyses: treatment assignment, disease burden, Gleason score (GS), World Health Organisation (WHO) performance status (PS), age and serum prostate specific antigen (PSA) obtained prior to androgen deprivation therapy (ADT) of the FTC to Abi781. **C**. Spearman correlation plot of 57 (continuous) signatures (detailed in Supplemental Table 2) included in the outcome analyses, ordered by first principal component in M0 cohort (matched in M1 cohort) and annotated for signature biology group (a) and cancer type the signature was developed in (b). M0 cohort in the bottom-left triangle and M1 in the top-right triangle. Size of circle represents level of correlation. M0N0: high-risk localized node-negative, M0N1: high-risk localized node-positive, M1LV: metastatic low-volume, M1HV: metastatic high-volume, RT: radiation therapy, AAP: abiraterone acetate and prednisolone.

Of 1917 patients (full-trial cohort) enrolled between 15^th^ November 2011 and 17^th^ January 2014, 1824 (95%) consented to participate in molecular ancillary studies (Supplemental Figure 1). We retrieved formalin-fixed, paraffin-embedded diagnostic tissue (biopsies or trans-urethral resections of the prostate, TURPs) from 1298 patients (71%) from 103 (of 111) UK clinical trial sites (Supplemental Figure 2) and centrally reviewed them for Gleason score, tumor content and tumor lymphocyte infiltration. Samples from 1014 patients (78%) had sufficient tissue and tumor purity. Between 6^th^ March 2019 and 10^th^ March 2022, mRNA was extracted from freshly-cut, 5 or 10-μm sections and transcriptome-wide expression values were generated on 831 (82%) patients. From these, 781 (94%, hereafter referred to as the Abi781 cohort) had data passing quality control (94% transrectal biopsies [49% M1, 51% M0], 6% TURPs [64% M1, 36% M0], Figure 1A): clinical characteristics of these patients were representative of the full-trial cohort (Figure 1B, Supplemental Table 1).

Most patients in the full-trial and Abi781 cohorts were starting their primary treatment for synchronous metastatic or high-risk localized disease (95% and 97% respectively), the rest were starting long-term ADT for relapse after primary radical therapy. 84% of localized disease (none of the M1) patients were planned for radical radiotherapy to the prostate (and pelvic nodes if clinically indicated). Of the patients with metastatic disease in Abi781, 40% were classified as low-volume, 55% high-volume and data was missing for 5% (N=19): these were excluded from analyses including this variable. Of the high-risk localized disease patients, 61% were N0 and 39% were N1. Luteinizing hormone-releasing hormone agent (LHRHa, 72% agonist, 28% antagonist) was administered prior to biopsy in 58 patients (7.4% of Abi781) with the majority being M1 high-volume (4 [2%] M0N0, 2 [1%] M0N1, 6 [4%] M1 low-volume, 43 [20%] M1 high-volume, 3 [16%] M1 unknown). The sub-set of patients with tumor tissue retrieved before LHRHa (hereafter referred to as the Abi723 cohort) had lower presenting serum PSA (median: 50ng/mL [IQR: 19–122] versus 56ng/mL [21–149]) but similar age, Gleason score and disease burden compared to the full-trial and Abi781 cohorts (Supplemental Figure 3). The median time from biopsy to randomisation was 77 days (IQR: 59–98) for newly-diagnosed patients (N=755) and 1013 days (699–1635) for relapsed patients (N=26). This trial was closed to further follow-up on 30^th^ November 2021 and outcome analyses were performed on data from the final lock (3^rd^ July 2022, reported elsewhere^19^, Supplemental Figure 4). Median follow-up was 94 months (IQR: 84–97).

### Transcriptome signature overview

We used pre-computed and locked signatures from the Decipher Genomics Resource Information Database (GRID) including 100,512 prostate cancers (16% low, 31% intermediate and 6% high National Comprehensive Cancer Network [NCCN] risk at diagnosis; 42% after prostatectomy; described previously^5^), optimized using the code or gene data from the original publications (Supplemental Table 2). We selected 59 signatures as a summary of expression data representative of biological pathways we hypothesized as relevant to prostate cancer. Of these signatures, 26 were developed on prostate cancer samples and 33 were developed using other tumor types (pan-cancer or exclusively bladder, breast, gastric, melanoma or renal cancer). We categorized these signatures as providing information on androgen receptor (AR) or steroid receptor signalling, including AR splice variant ARv7^5,20–27^ (15/59 signatures), immune infiltration and tumor microenvironment^23,28–33^ (N=14/59), prostate subtyping into neuroendocrine or basal/luminal phenotypes^4,24,34–38^ (N=8/59) or ERG fusion events, loss of PTEN or TP53, or *SPOP* mutations^39–44^ (N=7/59), hallmarks of cancer^23^ (N=7/59), DNA repair pathways^8,45–47^ (N=5/59) or risk-of-progression models^6,48,49^ (N=3/59). A continuous score (57/59 signatures) or categorical group (2/59) was ascribed to every tumor for each signature. Signatures related to androgen receptor signalling were correlated with each other and anti-correlated with signatures representing a neuroendocrine phenotype (Figure 1C).

ERG and PTEN have been extensively studied in prostate cancer. Our ERG transcriptome signature was shown to have a high AUC (0.96 [0.93–0.98]) with IHC distinguishing ERG-fusion positive from negative^39,50^. The PTEN_Liu signature (developed using prostate cancer data from The Cancer Genome Atlas) showed 0.89 specificity and 0.77 sensitivity in an independent dataset to identify genomic loss of PTEN^41^. We compared this signature with a validated IHC test^51,52^ and whilst we identified a higher proportion of low PTEN_Liu tumors in the IHC-null group, we noted differences between testing approaches (AUC 0.80 [0.76–0.85], Supplemental Figure 5).

### Signature distribution in patients split by disease burden

**Figure 2:**
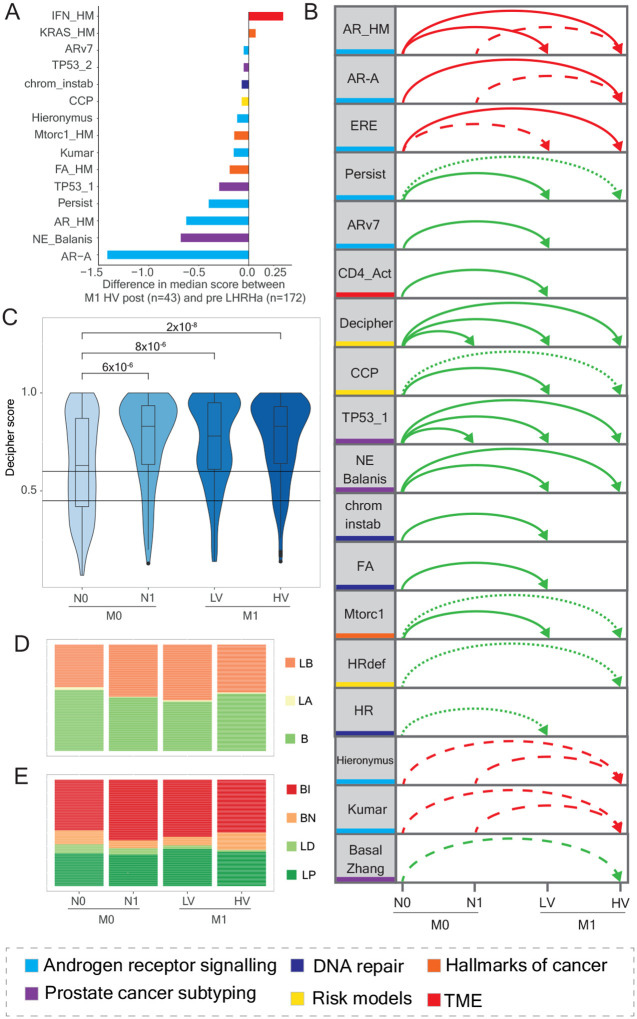
Signature score distribution (continuous or categorical) by disease burden. **A**. Diverging barplots of 15 signatures which are statistically-significantly differently distributed (p<0.001) in M1 high-volume tumors collected after versus prior to LHRHa, ordered by difference in median signature score and coloured by signature biology (detailed in Supplemental Figure 7). **B**. Arc diagram of the 18 signatures whose scores are statistically-significantly different (p<0.001) by disease burden (M0N0, M0N1, M1 low-volume and M1 high-volume) in the Abi781* and Abi723* cohorts and denoting between which states they are different. Colored by signature biology and ordered by which cohort they are significant in (both, Abi723 then Abi781). Red lines indicate the score decreases with increasing disease burden (direction of arrow) whilst green lines indicate the score increases. Dotted lines represent significant associations in Abi723, dashed lines in Abi781 and solid lines in both Abi723 and Abi781. **C**. Box-violin plots showing the distribution by disease burden of Decipher scores in Abi723* and the Wilcoxon rank sum test p-values. Threshold for low (0.45) and high Decipher score (>0.60) shown. **D**. Stacked barplots showing the frequency distribution by disease burden in Abi723* of the three categories for PAM50 (basal [B], luminal A [LA] and luminal B [LB]). **E**. Stacked barplots showing the frequency distribution by disease burden in Abi723* of the four categories for PSC (basal immune [BI], basal neuroendocrine [BN], luminal differentiated [LD], luminal proliferating [LP]). LHRHa: luteinizing hormone-releasing hormone agonist or antagonist. M0N0: high-risk localized node-negative, M0N1: high-risk localized node-positive, M1LV: metastatic low-volume, M1HV: metastatic high-volume, PSA: prostate serum antigen. *excluding 19 M1 cases where disease burden is unknown.

As a subset of patients had received LHRHa prior to sampling, we compared the signature score distribution (for continuous signatures) of tumors from M1 high-volume patients acquired post- versus pre-LHRHa (N=43). As expected, signatures that were statistically-significantly different (N=15/57, Wilcoxon rank sum test, p<0.001) included signatures related to AR and other steroid signalling (6/14) but differences were also observed for hallmark signalling pathways (3/7), prostate cancer subtyping (3/13), tumor microenvironment (1/11), DNA repair (1/5) and risk-of-progression models (1/3, Figure 2A, Supplemental Figure 6). We then evaluated differences in the distribution of signatures across disease burden. Noting the effects of testosterone suppression, we did this in both the Abi781 and Abi723 cohorts. We identified 15/57 continuous signatures (27%) that showed statistically-significant differences (adjusted p<0.001) in Abi723 and an additional three signatures in Abi781 (18 in total, with 13 showing statistically-significantly different distributions in both, Figure 2B, Supplemental Table 3). The most commonly observed differences were between M0N0 and low-volume (13/18) or high-volume (13/18) metastatic disease. We found no differences between M0N1 and M1 low-volume nor between metastatic volume groups. Decipher (Figure 2C) and a signature of TP53 loss had statistically-significant differences when comparing N0 and N1 high-risk localized cases.

We subtyped tumors into basal or luminal using prediction analysis of micro-array 50 (PAM50) and prostate subtyping classifier (PSC). PAM50 was developed in breast cancer and associated with post-prostatectomy outcomes (luminal B patients had worse outcomes^4^). In both Abi781 and Abi723, 2% of patients classified as luminal A (Figure 2D, Supplemental Figure 7A, B) compared to ~43% in low-risk, localized disease patients included in the Decipher GRID, (Chi-square test of independence, p<0.001, Supplemental Figure 7C). The distribution of PAM50 categories was similar across disease burdens. We developed PSC using prostate-specific gene variability in the Decipher GRID to identify luminal and basal sub-types and found that in advanced prostate cancer, 53% were categorized as basal immune (BI), 11% basal neuroendocrine-like (BN), 31% luminal proliferating (LP) and 5% luminal differentiated (LD, Figure 2E, Supplemental Figure 7D, E). In contrast to PAM50, we observed a difference in PSC categories with increasing disease burden, namely luminal differentiated was more common in M0N0 patients (8% versus 2–6% in other disease burden groups, Chi-square test of independence, p<0.001) that was consistent with a higher prevalence in lower-risk localized tumors from the Decipher GRID (55%, Supplemental Figure 7F). On unsupervised clustering of median (continuous) signature scores by metastatic state and by PSC and aligned PAM50 categories, both PSC basal neuroendocrine-like and PAM50 basal sub-types had higher scores for neuroendocrine and lower scores for AR-related signatures (Supplemental Figure 8).

### Primary outcome analyses

All our outcome analyses were pre-defined in a statistical analysis plan (signed 21^st^ March 2022, appendix). We defined a single signature for primary prognostic analysis: Decipher^6^ and four signatures (AR-A^5^, PAM50^2^, PSC^37^ and Decipher^53^ in this order using a closed hierarchical test) for primary predictive analyses (i.e., interaction of signature with treatment effect). These signatures were selected as they showed associations with outcome in localized prostate cancer^4–6^ or in metastatic sets^54,55^, as well as representing important prostate cancer biology and capturing distinct groups of patients (Supplemental Figure 9). We found no statistically-significant differences (p<0.001) in signature distribution by treatment assignment (Supplemental Table 4). In our primary prognostic analysis (on Abi781), we tested each clinical and pathological feature in a univariable model (Supplemental Table 5) and then built a multi-variable model that included Decipher score as a continuous variable. We found that every 0.1 unit increase in Decipher score was associated with a significant worsening of both overall survival in M1 patients (HR 1.18 [95% CI, 1.09–1.27], p=5×10^−6^) and metastasis-free survival in M0 patients (HR 1.20 [1.10–1.31], p=2×10^−5^, Table 1). We observed similar results in Abi723 (Supplemental Table 6).

**Figure 3:**
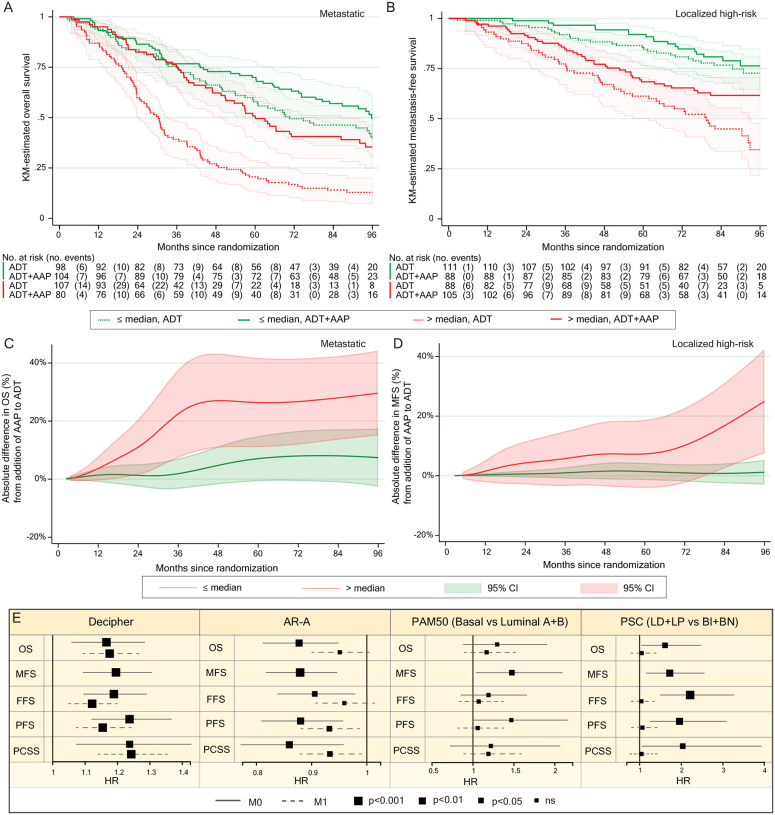
Primary and secondary outcome analyses. **A**. Kaplan-Meier plot of overall survival in patients with metastatic disease in Abi781 receiving either ADT or ADT + AAP split by Decipher score above or below median (0.77 on scale of 0–1). **B**. Same as for A but showing localized disease patients and metastasis-free survival. **C**. Line plot showing the absolute difference in overall survival (as a percentage) of addition of AAP to ADT in patients with metastatic disease in Abi781* split by Decipher score above or below median with 95% confidence intervals adjusted for clinical and pathological variables. **D**. Same as for C but showing localized disease patients and metastasis-free survival. **E**. Prognostic forest plots of Decipher, AR-A, PAM50 and PSC with primary and secondary outcomes by metastatic state and adjusted for clinical and pathological variables, scaled to fit the range of 95% CI for each signature for Abi781*. Full line for M0 and dashed line for M1 with size of hazard ratio square relative to level of statistical significance of association. ADT: androgen deprivation therapy, AAP: abiraterone acetate and prednisolone, M0: high-risk localized, M1: metastatic, HR: hazard ratio, CI: confidence interval. *excluding 18 M1 cases where disease burden is unknown, three where Gleason score is unknown and one where both are unknown.

We then performed survival analysis on Abi781 split by median Decipher score (0.77, Figure 3A, B) and explored differences in the absolute benefit of adding abiraterone to ADT (Figure 3C, D). M1 patients with higher Decipher scores had greatest benefit from addition of abiraterone to ADT in the first 36 months that then reached a plateau. In contrast, fewer events in high-risk localized disease patients in the first 72 months resulted in no apparent difference in the benefit of adding abiraterone to ADT. This is characterized after longer follow-up by a notable divergence in absolute benefit gained from abiraterone for patients with higher Decipher scores and limited benefit in patients with lower Decipher scores.

No signature met the pre-determined significance threshold for predictive analysis (Supplemental Table 7).

### Secondary outcome analyses

In our secondary analyses we confirmed a strong and statistically-significant association of the Decipher score with all secondary outcome measures (Abi781, Figure 3E; Abi723, Supplemental Table 6). In M0 disease, AR-A was strongly prognostic (protective) for all endpoints, with increasing AR-A score predicted to have longer metastasis-free (HR per unit increase 0.88 [0.82–0.94], p=0.0008), overall (HR 0.88 [0.81–0.95], p=0.001), progression-free (HR 0.88 [0.81–0.96], p=0.004), and prostate cancer-specific (HR 0.86 [0.77–0.96], p=0.008) survival. In M1 patients, AR-A was not associated with overall survival (HR 0.95 [0.90–1.01], p=0.10) in Abi781 and weakly in Abi723 (HR 0.93 [0.87–0.99], p=0.03). Increasing AR-A also showed weak associations with better outcome for secondary end-points, slightly more strongly in Abi723 (Figure 3E, Supplemental Table 6). Overall, the association with better outcomes of AR-A in localized high-risk patients was less evident for metastatic disease although this may be confounded by testosterone suppression prior to biopsy in a sub-set of metastatic patients.

For analyses of associations with outcome by PAM50, we used PAM50-basal as the reference category and, in keeping with prior studies^4,54^, found a weak association in both Abi781 and Abi723 with shorter metastasis-free survival in M0 compared to combined luminal sub-groups (Abi781, HR 1.47 [1.03–2.08], p=0.03; Abi723 HR 1.52 [1.06–2.17], p=0.02). No other significant associations were observed in either Abi781 or Abi723.

For PSC, we first combined luminal differentiated and luminal proliferating sub-groups as the reference category and compared to combined basal neuroendocrine-like and basal immune sub-groups: we found PSC basal sub-types were associated with shorter metastasis-free survival in M0 patients (HR 1.77 [1.20–2.62], p=0.003). The same was observed for failure-free (HR 2.19 [1.49–3.22]; p=0.00003) and progression-free survival (HR 1.96 [1.25–3.07]; p=0.002), with similar results in Abi723 (Supplemental Table 6). We then tested associations in the four groups separately, with basal immune as the reference and confirmed these had worse outcomes than either basal neuroendocrine-like, luminal differentiated or luminal proliferating in M0 (p=0.0004, respectively for metastasis-free survival HR 0.45 [0.23–0.91], 0.22 [0.07–0.71] and 0.60 [0.40–0.90]) but not in M1 (p=0.93, respectively for overall survival HR 0.89 [0.58–1.36], 1.00 [0.40–2.51] and 0.92 [0.68–1.24], Supplemental Figure 10).

### Further analyses of transcriptomic classifiers and associations with outcome

**Figure 4:**
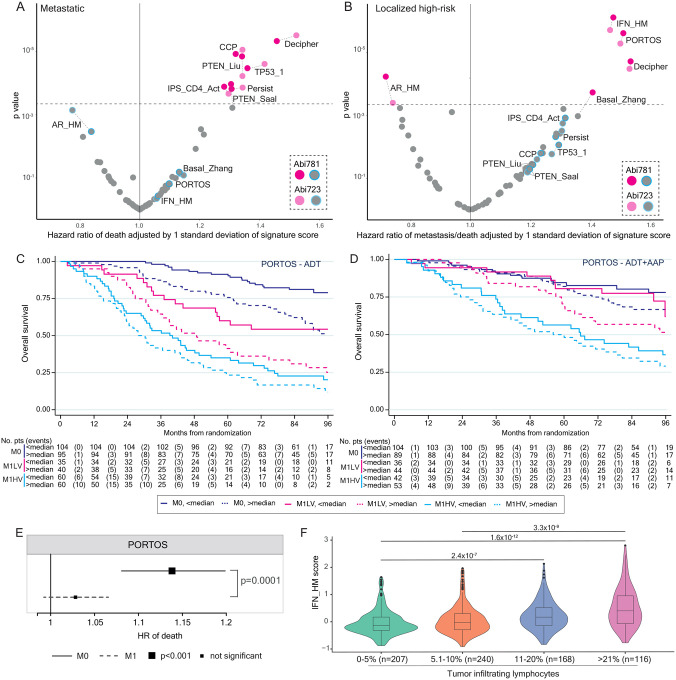
Exploratory outcome analyses. **A.** Scatter plot of hazard ratios (adjusted by one standard deviation of signature score) and p values resulting from prognostic testing adjusted for clinical and pathological variables of overall survival in 57 signatures (with continuous scores) in patients with metastatic disease*. Dots representing statistically significantly prognostic (p<0.00045) signatures in Abi781 and Abi723 are colored in dark pink and light pink respectively. Signatures which are prognostic in M0 are colored in the outer circle in Abi781 and Abi723 in blue and light blue respectively. Signatures which are not prognostic in Abi781 or Abi723 are colored in grey. Dotted lines connect values for the same signature. Dashed line marks p=0.00045. **B**. As for A but in M0 patients. **C**. Kaplan-Meier plot of overall survival in patients** receiving ADT split by metastatic state (M0 and M1) and PORTOS score (above or below median in Abi781: −0.31) with M1 patients further split by disease volume (low and high). **D**. As for E but for patients** receiving ADT+AAP. **E**. Forest plot of PORTOS, indicating the p value for interaction with metastatic state, and hazard ratio, 95% CI and p value in metastatic states for Abi781*. Full line for M0, dashed line for M1 with size of hazard ratio square relative to level of statistical significance of association. **F**. Box and violin plot of IFN_HM score split by tumor infiltrating lymphocytes (four groups: 0–5%, 5.1–10%, 10.1–20%, >20.1%) with added p values for significance. N=731, does not include 43 cases which were TURPs and seven biopsies that could not be assessed for technical reasons. HR: hazard ratio, M0: high-risk localized, M1: metastatic. ADT: androgen deprivation therapy, AAP: abiraterone acetate and prednisolone. *excluding 18 M1 cases where disease burden is unknown, three where Gleason score is unknown and one where both are. **excluding 19 M1 cases where disease burden is unknown.

We expanded our prognostic and predictive testing to all other signatures (N=55), none of which had been previously tested in advanced prostate cancer. We here focus on signatures showing the strongest associations with outcome in both Abi781 (p<0.00045, selected due to the conduct of multiple tests equivalent to 110 permutations) and Abi723 (Figure 4A, B, Supplemental Table 8). In patients with metastatic disease, we observed a statistically-significant association with overall survival for signatures that have been previously proposed to be prognostic on single-gene testing, namely loss of PTEN, using the prostate-specific PTEN_Liu^41^ signature (Abi781: HR 1.08 [1.05–1.12], p=1.4×10^−5^; Abi723: HR 1.08 [1.04–1.12], p=5.9×10^−5^) or TP53 loss (TP53_1^43^, Abi781: HR 1.13 [1.06–1.19], p=3.3×10^−5^; Abi723: HR 1.15 [1.07–1.22], p=2.4×10^−5^). Additionally we confirmed a strong association with overall survival and cell cycle progression^49^ (Abi781: HR 1.29 [1.16–1.43], p=1.2×10^−5^; Abi723: HR 1.30 [1.17–1.46], p=8.7×10^−6^) and Persist, a signature developed to represent cells that persisted after therapy^25^ (Abi781: HR 1.97 [1.39–2.81], p=0.0002; Abi723: HR 2.12 [1.44–3.12], p=0.0001). We also observed a previously undescribed association of survival with a profile representative of increased CD4 cell activity^28^ (IPS_CD4_Act, Abi781: HR 1.23 [1.11–1.36], p=0.0001; Abi723: HR 1.24 [1.11–1.38], p=0.0002). A signature of PTEN loss developed in breast cancer (PTEN_Saal^40^) was statistically-significant in Abi781 but the risk of death estimates in Abi723 were below our threshold.

In high-risk localized disease, in keeping with our secondary outcome analysis on AR-A, we observed a protective effect for increased AR signalling, measured by the AR hallmark signature (AR_HM^23^, Abi781: HR 0.93 [0.91–0.97], p=5.9×10^−5^; Abi723: HR 0.94 [0.91–0.97], p=0.0004) and a worse outcome for basal sub-types using a modified classifier (Basal_ Zhang^34^) in Abi781 but not in Abi723. Notably we also observed statistically-significant associations with worse outcome with increased interferon-alpha response signalling (IFN_HM^23^, Abi781: HR 1.07 [1.04–1.09], p=7.9×10^−7^; Abi723: HR 1.07 [1.04–1.09], p=2.0×10^−6^) and Post-Operative Radiation-Therapy Outcomes Score (PORTOS^8^, Abi781: HR 1.12 [1.07–1.18], p=2.5×10^−6^; Abi723: HR 1.12 [1.07–1.18], p=5.3×10^−6^, Figure 4B).

There was no statistically-significant treatment interaction between any signature and addition of abiraterone to ADT (Supplemental Table 9).

### Dynamic associations with survival of PORTOS across metastatic states

We were intrigued that PORTOS was strongly associated with outcome in high-risk localized but not patients with metastatic disease, especially given we found no difference in signature score distribution across disease burden nor change with prior LHRHa (Figure 2A, Supplemental Figure 11). To visualize this result, we generated Kaplan-Meier curves of patients dichotomized by median PORTOS (−0.31) and metastatic state. Given low-volume M1 patients benefit from radiotherapy to the prostate but high-volume do not^56^, we further split patients by metastatic volume. We observed a significant difference in survival in M0 patients (HR per unit increase in PORTOS score 1.14 [1.08–1.20], p=1.5×10^−6^) but no statistically-significant difference in survival in neither low-volume (HR 1.06 [1.00–1.14], p=0.07) nor high-volume (HR 1.02, [0.98–1.07], p=0.39) patients (Figure 4C, D). Although not pre-defined in our statistical analysis plan, we then proceeded to test for and identified a significant interaction of the associations of PORTOS with overall survival and presenting metastatic state (interaction HR: 0.89, [0.84–0.95], p=0.0001, Figure 4E, Supplemental Table 10).

### Tumor immune axis and long-term survival

We further investigated interferon signaling (IFN_HM), which showed the most statistically-significant association with worse outcome in high-risk localized disease. We confirmed a significant association with overall survival (HR 1.07 [1.04–1.10], p=5×10^−6^) but found no association in patients with metastatic disease (HR 1.01 [0.99–1.03], p=0.35, test for interaction of overall survival with metastatic state HR 0.94 [0.91–0.97], p=0.0001, Supplemental Table 10). The IFN_HM signature captures a wide range of variation in the expression of genes associated with interferon signalling and showed no difference in distribution across disease burden (Supplemental Figure 12). It also weakly correlated with other immune-related signatures, including IPS_CD4_Act (summarized in Figure 1C) that was associated with survival in patients with metastatic disease. We performed pathway analysis on pan-transcriptome genes showing the strongest correlation with IFN_HM signature genes and identified enrichment of JAK-STAT1 signalling (family-wise error rate=3×10^−16^, Supplemental Table 11). This finding is in keeping with activation of a type 1 interferon response in tumors with increasing IFN_HM^57^. We then posited that tumors with increased IFN_HM would have more tumor infiltrating lymphocytes: as part of our sample processing work-flow, we assigned all tumors to one of four categories based on the proportion of cells in a tumor region that were lymphocytes. We discovered strong and significant associations between tumoral areas with ≤5% lymphocyte infiltration compared to ≥10–20% or >20% and IFN_HM (respectively p=2.4×10^−7^, p=1.6×10^−12^, Figure 4F). We then used CD4-CD8-Foxp3 triple-staining IHC on a slide sequential to the H&E in a sub-set of related STAMPEDE cases (N=425) and confirmed our H&E-based tumor infiltrating lymphocyte counts were representing mostly T cells, equivalent to the sum of CD4+Foxp3- (median proportion of total in each case: 50%, IQR: [45–58%]) plus CD8+Foxp3- (45% [40–50%]) plus CD4+Foxp3+ (3% [1–5%]) T cells (Supplemental Figure 13). As denoted by the narrow range of distributions of each cell type, the ratios of CD4+:CD8+ cells were uniform across tumors regardless of total lymphocyte count, with no noticeably different proportion of either cell type across sub-groups of tumors.

## DISCUSSION

In this study we confirmed that expression-based signatures can provide clinically-relevant prognostic information in advanced prostate cancer independent of prognostic clinical variables, including Gleason score and disease burden. We benefited from the STAMPEDE cohort design that included both high-risk localized disease patients receiving finite adjuvant therapy and patients with metastatic disease treated to progression and death: this allowed evaluation of outcome in both states. Next-generation sequencing has been extensively used on fresh samples but routine, clinical formalin-fixed prostate biopsies are associated with a high-failure rate and significant variability; our microarray-based test is clinical grade, robust and provided data on the majority of cases, offering a clear path to clinical implementation.

Across signatures tested, the Decipher signature was the only one associated with outcome in both metastatic and high-risk localized disease. The relative benefit of adding abiraterone to ADT was consistent across molecular sub-types, but the absolute benefit for addition of abiraterone was substantially greater in patients with a higher Decipher score. This is most clinically relevant in high-risk localized disease patients for whom other competing risks of death may outweigh the absolute benefit yielded by hormone intensification. In metastatic disease, Decipher identified patients at increased risk of death within 32 months and could be considered when selecting patients for more intensified treatment. We dichotomized our cohort by the median Decipher score and fortuitously, the equivalent value in recently-acquired samples is undergoing evaluation in prospective trials in localized disease (NCT04513717). Further work will now be required to build prognostic models for clinical implementation.

Our study is consistent with prior work using single-gene assays, such as the absence of prognostic associations for classifiers of AR-v7^58^ and ERG^59^ in contrast to loss of PTEN^60^ and TP53^61,62^ that have been previously associated with worse prognosis. The latter observations suggest clinical utility for using transcriptome classifiers to define clinically-relevant PTEN-loss biology that could be therapeutically targeted, potentially improving on current tests using genomic alterations or IHC^63^. Transcriptome-wide analysis also provides the opportunity to improve prognostic accuracy by integrating signatures representative of distinct molecularly-relevant pathways such as those measuring cell proliferation^49^ (CCP) or pre-therapy resistant cells (Persist) that were developed in *de novo* metastatic prostate cancer or pre-clinical metastatic prostate cancer models respectively.

In high-risk localized disease, an increase in type 1 interferon signaling that associates with increased tumor lymphocyte infiltration was detrimental. Signatures for interferon alpha and gamma signaling were strongly correlated so interferon alpha was chosen for prognostic testing. Several signatures representing the immune microenvironment were correlated: thus the complex interplay of the tumor-immune axis and whether specific interferon types play differential roles requires further study. Intriguingly, studies in other cancer types identified increased tumor interferon and IL6-JAK-STAT signaling prior to radiation as associated with poorer response^64^ in keeping with our observation of worse outcomes for patients treated with prostate radiotherapy. We also found that the basal immune PSC sub-type have worse outcomes than all other sub-types in M0 disease, consistent with parallel work on lower-risk cohorts (manuscript under review).

We also present evidence of differential effects on outcome for distinct biological processes based on presence or absence of metastatic disease on conventional scans at time of first presentation (managed by different treatment paradigms).This is not reflected in differences in signature distribution across metastatic states. The finding was strongest for PORTOS and was serendipitous given this signature was developed in patients receiving adjuvant or salvage radiotherapy post-prostatectomy^8^ and had not been previously tested in patients presenting with metastatic disease. Given radiotherapy to the primary is effective for low-volume patients only, we present Kaplan-Meier curves that split metastatic patients by disease volume and found a marginal association with outcome in low-volume disease. In this context, low-volume M1 patients may represent an intermediate clinical stage between M0 and M1 high-volume. The premise that the impact on clinical outcomes of tumor biology is metastatic state context-dependent is supported by clinical evidence of differential benefit to radiation or docetaxel across disease states^56,65^.

There are limitations to our study. Firstly, the collection and analysis of tissue was optional and initiated after completion of accrual to the trial. The majority of participating centres contributed samples and we included a large and consistent number of patients in each disease burden group, which minimises undetected biases related to tumor availability. Secondly, clinical practice may require immediate initiation of hormone therapies, including first-generation AR antagonists (most commonly bicalutamide) or LHRHa, most commonly in patients with M1 high-volume disease who are more likely to be symptomatic. To ensure our cohort was representative of the full-trial cohort and routine clinical practice, we included all 781 patients in our primary analysis but limited our conclusions to findings that were consistent in Abi723. Our conclusions are therefore relevant to clinical implementation and account for the effect of prior treatment, including highlighting the limitation of testing AR-based signatures in metastatic patients. Thirdly, given our analysis was transcriptome-wide, there were risks of over-fitting. We accounted for this by limiting to 59 signatures and we pre-specified our analyses before any testing of associations with outcome were undertaken (solely by trials unit statisticians).

As our data has been generated in a CLIA-certified lab using a clinical grade test, our signatures are robust, linked to high-quality, prospectively-collected long-term follow-up and will be reliably comparable across other datasets. We will make our data available as a resource for the community for further testing of biological hypotheses. In our study, transcriptomes were not used to select treatment and our data cannot therefore be considered Level 1 evidence for patient selection for treatment. Studies that prospectively stratify advanced prostate cancer patients based on tumor expression profiles are now required. In conclusion, in this large ancillary study of the abiraterone phase 3 trial conducted within the STAMPEDE platform protocol, we showed that expression classifiers measured at diagnosis from routinely-acquired primary tumors can characterize advanced prostate cancers into clinically-useful groups and allow interrogation of molecular pathways that define the *in vivo* interactions of tumor biology and clinical progression.

## METHODS

### Patient Cohort

Patients were randomized in the abiraterone trial of the STAMPEDE platform protocol (Medical Research Council PR08, NCT00268476, EUDRACT: 2004–000193-31, ISRCTN: ISRCTN78818544)^9^ that was sponsored by the Medical Research Council (up to 2013) and is now sponsored by University College London. Briefly all patients had histologically-confirmed prostate adenocarcinoma and were eligible if they had metastatic disease confirmed on conventional whole-body computed tomography and technetium bone scans or localized, high-risk disease that was node-positive or, if node-negative, had at least two of tumor stage category T3/4, PSA ≥40ng/ml, Gleason score 8–10. Prostate serum antigen prior to LHRHa was obtained up to 6 months before randomization. Gleason score, age, stage, WHO performance status and metastatic state and disease burden, namely M0 N0, M0 N1 or M1, were recorded by clinical sites and accessed from the STAMPEDE trial database. Metastatic burden was further classified into high or low volume based on the presence of visceral metastases or ≥4 bone metastases with ≥1 outside the vertebral bodies or pelvis. Date of biopsy was obtained from histopathology reports, date of start of LHRHa was recorded at time of randomization. Patients who started LHRHa more than five days before sample acquisition were excluded from the Abi723 cohort.

### Study Approval

These analyses were performed as part of the STRATOSPHERE (STratification for RAtional Treatment-Oncomarker Pairings of STAMPEDE patients starting long-term Hormone treatment) consortium protocol for molecular studies on tumors collected from patients treated in the STAMPEDE trial, approved by an independent Research Ethics Committee (REC18/LO/1235) and the STAMPEDE Trial Management Group and Trial Steering Committee. All patients signed informed consent to participate in the STAMPEDE trial and for use of their tissue for research (/REC04/MRE07/35).

### Sample retrieval and processing

Tissue blocks were retrieved from STAMPEDE trial sites when requested by the Medical Research Council Clinical Trials Unit at UCL and centralized at the Wales Cancer Biobank, where all identifying details were removed. Tissue blocks were transferred to the UCL Cancer Institute (London, UK) for processing and where sample type was recorded. Central pathology review of freshly-cut H&E slides (LM, DMB, Supplemental Table 12), including categorization of infiltrating tumor immune cells (tumor infiltrating lymphocytes [TILs]) following the International Immunooncology Biomarkers Working Group methodology^66^, was performed. TILs were counted within the tumor cell compartment only and separately for each diagnostic biopsy core assessed. A semiquantitative analysis was performed, with discrete values given according to the proportion of infiltrating lymphocytes in the tumor cell compartment (0–80%). When integrating with transcriptomic signature scores, the highest TIL count for each patient was used. Trans urethral resections of the prostate (TURPs) were not evaluated and samples with technical artefacts were excluded. CD4-CD8-Foxp3 IHC triple immunostaining using a previously-described clinical test^67^ was performed on a second freshly-cut 5 or 10μm slide (sequential to the H&E section) from a related cohort and provided orthogonal validation of TIL counts assessed on the H&E slides (Supplemental Figure 12). The tumor block containing the index core (selected using the following hierarchy: the highest primary Gleason score, the highest secondary Gleason score and the longest tumor length) was sectioned onto glass slides and analysed for expression of 1.4 million genome-wide features (including all known genes and most of the non-coding RNAs) in the Veracyte CLIA lab (San Diego, CA, USA) using high-density oligonucleotide arrays (Affymetrix Human Exon 1.0 ST, ThermoFisher, Santa Clara, CA, USA). Expression data was quality controlled by visualizing density and relative expression plots before and after background correction and quantile normalization using the affy^68^ package in R. Multi dimensional scaling (MDS) plots were used to inspect for possible batch effects and corrected using psva14 if needed. Data normalisation was performed using Single Channel Array Normalisation, where each sample was compared to itself using control probes from the array^69^. The Decipher GRID including expression profiles of >100,512 cancers from prospective clinical use of the Decipher test and retrospective studies of institutional cohorts was used to check the clustering solution and its analytical validity. Lab researchers had no access to clinical data other than pseudo-anonymized local pathology reports.

### Transcriptomic signature analyses

Signature scores were derived for all cases using signatures contained in the GRID. Spearman correlation coefficients were calculated using R^70^ (v1.4). Normalized gene expression values for over 46,000 transcripts were also obtained. Four signatures were selected for the primary and secondary analyses, namely AR-A, PAM50, PSC and Decipher. PAM50 was split into three classes, basal, luminal A and luminal B. PSC was also split into four classes: basal immune (BI), basal neuroendocrine-like (BN), luminal proliferating (LP) and luminal differentiated (LD). To perform quantile matching for PSC, the subset of the Decipher GRID classified as NCCN very high risk (1,412 samples) was used as the reference population. The expression data of the STAMPEDE biomarker analysis cohort was then quantile matched to that from the reference population and PSC were calculated on the matched expression data. Statistical tests assessing the signature score differences between metastatic states, disease burden, treatment arms, PAM50 and PSC categories, as well as assessing IFN_HHM signature score differences between TIL frequency groups were performed using a Wilcoxon rank-sum test. Statistical tests assessing the difference in group size between PAM50 categories between NCCN low and our biomarker cohort, as well as those assessing the prevalence of PSC groupings by metastatic state were performed using a Chi-square test of independence. Both performed using R. Data visualisation also performed using R.

### Statistical outcome analyses

The statistical analyses were pre-stated in the statistical analysis plan (appendix). The unblinded trials unit statisticians (CB, LMu, PDM) had sole access to the clinical trial data for these analyses. The primary outcome measures were as defined previously, namely overall survival (OS, time from randomisation to death from any cause) for patients with metastatic disease and metastasis-free survival (MFS, time from randomisation to first of radiologically-confirmed metastatic disease progression or death from any cause) for high-risk localized disease patients. Secondary outcomes were failure-free survival (FFS, time from randomisation to first of biochemical failure, local, lymph node or distant metastases progression, skeletal-related event confirmed as disease progression or death from prostate cancer); progression-free survival (PFS, as FFS but excluding biochemical failure); and prostate cancer-specific survival (PCSS, time from randomisation to death classified as due to prostate cancer). Patients with no event recorded for an outcome were censored at the last time they were known to be event-free.

All assessments of the association between each signature of interest and the hazard of the relevant outcome were performed using separate Cox models adjusted for treatment allocation (ADT vs ADT and abiraterone), age at randomisation (years), WHO performance status (0 vs 1 or 2), regular aspirin or NSAID use (no vs yes, included as a minimisation factor in the STAMPEDE platform protocol due to concurrent trials testing celecoxib), PSA prior to start of ADT (ng/ml, log-transformed), Gleason score assessed by local pathologist (categorical), and disease burden (M0 N0 vs M0 N1 or M1 low-volume vs M1 high-volume). Models were stratified by protocol-specific time period at time of randomisation, defined by other arms recruiting to the trial or changes to standard of care. Analyses of predictive utility included a term for the interaction between treatment allocation and genomic signature. Likelihood ratio test p-value was used to assess evidence of predictive or prognostic effect. All tests are presented as two-sided, with 95% confidence interval (in brackets) around the estimated treatment or prognostic effect. Checking of the proportional hazards assumption was performed using a global Grambsch-Therneau test with log-transformed time. Kaplan-Meier curves, using the KMunicate format^71^ where appropriate, were used to illustrate survival in different patient groups.

Sensitivity analyses of the main predictive / prognostic tests used alternative models that included data from patients with relapsing disease at baseline. All analyses were repeated on Abi723 to study potential impacts of prior hormone therapy.

Exploratory testing for evidence of a different association between signatures of and survival outcome for patients by metastatic state at baseline (M0 and M1) was performed using Cox regression models. These were adjusted for the same covariates as other prognostic models, but included a binary indicator corresponding to whether a patient had M0 or M1 disease instead of the 4-category ‘disease burden’ covariate, plus an interaction term between this indicator and the variable representing the biomarker. The likelihood ratio test p-value associated with this interaction term was used to assess the strength of evidence in favour of a different association between signatures and outcome according to baseline metastatic state.

Kaplan-Meier estimation of survival at specific time points was supplemented by standardized (population average) estimates from adjusted flexible parametric survival models (including randomisation time period as a covariate), implemented using the -predict, meansurv- postestimation command for the Stata package -stpm2.

### Differential gene expression analyses

The Spearman rank correlation coefficient between each gene included in the hallmark interferon alpha response (IFN_HM) pathway and all other genes was calculated separately in high risk localized and patients with metastatic disease. Genes which had q < 0.05 and Spearman rank correlation coefficient > 0.75 were included in pathway annotation, which was performed separately for up and downregulated genes and metastatic state. The Benjamini-Hochberg correction^72^ was applied to correct the p-values for multiple testing. The R package Biomart v2.42.1^73,74^ was used to map gene symbols to ENSG. PathwAX II^75^ was used alongside the KEGG database^76^ to annotate pathways.

## Supplementary Material

1

## Figures and Tables

**Table 1:** Primary outcome assessment of Decipher score.

	Metastatic			Localized high-risk		
	HR	95% CI	p value	HR	95% CI	p value
**Treatment**						
Allocated to abiraterone (vs ADT)	0.54	0.41–0.70	<0.001	0.61	0.43–0.87	0.006
	
**Disease Burden**						
M1 high (vs M1 low)	2.08	1.56–2.77	<0.001			
M0N1 (vs M0N0)				1.88	1.31–2.70	0.001
	
**Gleason Sum**						
Gleason ≤7	0.93	0.61–1.32	0.72	0.60	0.34–1.05	0.07
Gleason 8 (reference)	1.00			1.00		
Gleason 9	1.29	0.93–1.80	0.13	1.15	0.77–1.72	0.49
Gleason 10	2.51	1.25–5.07	0.01	2.76	1.14–6.70	0.03
						
**Decipher**						
continuous per 0.1 unit increase	1.18	1.09–1.27	<0.001	1.20	1.10–1.31	<0.001

Hazard ratios, 95% confidence intervals (CI) and p values for clinical and pathological variables that had a p<0.05 in the multivariable Cox model testing Decipher and survival. Model also included WHO performance score, nsaid use, age and PSA at randomization (detailed in Supplemental Table 5). Excludes 18 M1 cases where disease burden is unknown, three where Gleason score is unknown and one where both are.

## Data Availability

Normalized expression values are available following approval of requests. Linked metadata is available upon request and after de-identification following approval by the MRC CTU at UCL data access committee. Please contact the corresponding author or mrcctu.stampede@ucl.ac.uk for more information.

## Footnotes

APPENDIX List of STAMPEDE investigators Signed statistical analysis plan (SAP)
