# Supplementary material for: Clinical testing of transcriptome-wide expression profiles in high-risk localized and metastatic prostate cancer starting androgen deprivation therapy: an ancillary study of the STAMPEDE abiraterone Phase 3 trial
Source: Res Sq. 2023 Feb 8:rs.3.rs-2488586. Preprint. [Version 1] doi: 10.21203/rs.3.rs-2488586/v1 (PMC9934744; doi:10.21203/rs.3.rs-2488586/v1)
Supplement: 1 [file NIHPPRS2488586V1-supplement-1.pdf]

SUPPLEMENTAL FIGURE LEGENDS

Supplemental Figure 1: Full trial cohort to Abi781 diagram. Full trial cohort to Abi781 diagram, by metastatic state, indicating at what stage of sample collection patients were removed from Abi781.

Supplemental Figure 2: Comparison of number of cases available for retrieval by STAMPEDE clinical trial site and percentage retrieved for this study. Scatter plot of number of cases available for retrieval by STAMPEDE clinical trial site and percentage of cases retrieved from that site for inclusion in this study.

Supplemental Figure 3: Comparison of main clinical features for Abi781 and Abi723. Stacked barplots and box-violin plots comparing proportion or range of baseline clinical features that were adjusted for in prognostic analyses: treatment assignment, disease burden, Gleason score (GS), World Health Organisation (WHO) performance status (PS), age and serum prostate specific antigen (PSA) obtained prior to androgen deprivation therapy (ADT) of Abi781 and Abi723.

Supplemental Figure 4: Kaplan-Meier plots for Abi781. A. Kaplan-Meier plot of overall survival in patients with metastatic disease split by treatment arm. **B**. Kaplan-Meier plot of metastasis-free survival in high-risk localized disease patients split by treatment arm. Both analyses performed using the data lock from 3rd July 2022.

Supplemental Figure 5: Comparison of PTEN signatures with IHC benchmark. A. Violin plots of PTEN_Liu signature scores compared to IHC assessment. **B**. Violin plots of PTEN_Saal signature scores compared to IHC assessment.

Supplemental Figure 6: Statistically-significantly different ($p<0.001$) signature scores in metastatic high-volume cases prior to or following five days on LHRHa. Boxplots showing signature score distribution of metastatic high-volume cases in cases sampled prior to LHRHa and after more than 5 days of LHRHa treatment.

Supplemental Figure 7: Distribution of PAM50 and PSC groups by NCCN risk score from the GRID. A. Stacked barplots showing the frequency distribution in Abi781 and Abi723 of the three categories for PAM50 (Basal [B], luminal A [LA] and luminal B [LB]). **B**. Stacked barplots showing the frequency distribution across disease burden in the Abi781* and Abi723* cases of the three categories for PAM50 (Basal [B], luminal A [LA] and luminal B [LB]). **C**. Stacked barchart of PAM50 class distribution by NCCN risk group (low, favorable intermediate, unfavorable intermediate, high and very high.) **D**. Stacked barplots showing the frequency distribution in Abi781 and Abi723 of the three categories for PSC (Basal immune [BI], basal neuroendocrine [BN], luminal differentiated [LD], luminal proliferating [LP]). **E**. Stacked barplots showing the frequency distribution across disease burden in the Abi781* and Abi723* cases of the three categories for PSC (Basal immune [BI], basal neuroendocrine [BN], luminal differentiated [LD], luminal proliferating [LP]). **F**. Stacked barchart of PSC class distribution by NCCN risk group. *excluding 19 M1 cases where disease burden is unknown.

Supplemental Figure 8: Heatmaps of median values of 57 continuous signatures split by metastatic state, PSC and PAM50 category. A. Heatmap of median signature score values in the Abi723 cases for signatures included in outcome analyses (n=57) across PSC and PAM50 categories split by metastatic state annotated for signature biology group (Figure 1C) and plotted using usupervised clustering based on PSC scores and categories, matched in the PAM50 categories.

Supplemental Figure 9: Upset plot of four primary signatures and categorical binary groupings. **A**. Upset plot showing the count of combinations of binary categories of the four primary signatures by metastatic states and a matched stacked barplot showing the frequency of each combination by disease burden for Abi781*. **B**. Upset plot showing the count of combinations of binary categories of the four primary signatures by metastatic states and a matched stacked barplot showing the frequency of each combination by disease burden for Abi723*. *excluding 19 M1 cases where disease burden is unknown.

Supplemental Figure 10: Kaplan-Meier plots for overall survival by PSC category and treatment. A. Kaplan-Meier plot of overall survival in patients with localized, high risk disease split by PSC category (LD, LP, BI and BN) and treatment arm. **B**. Kaplan-Meier plot of overall survival in patients with metastatic disease split by PSC category (LD, LP, BI and BN) and treatment arm.

Supplemental Figure 11: PORTOS score distribution by disease burden. Violin plot of PORTOS score by disease burden in Abi781*.*excluding 19 M1 cases where disease burden is unknown.

Supplemental Figure 12: Heatmap of scaled gene expression for genes in the IFN_HM score in Abi781 ordered by IFN_HM score. Disease burden, IFN_HM score and sample type annotated.

Supplemental Figure 13: Proportion of tumor infiltrating lymphocyte subtypes determined using IHC for CD4, CD8 and Foxp3 in related STAMPEDE cohort (N=425). **A**. Stacked barplot in descending percentage of tumor infiltrating lymphocytes including proportion of CD4+Foxp3-, CD8+Foxp3-, CD4+Foxp3+ cells determined using IHC. **B**. Stacked barplot of groups of TIL frequency (0-5%, 5.1-10%, 10.1-20% and >21%) including proportion of CD4+Foxp3-, CD8+Foxp3-, CD4+Foxp3+ cells determined using IHC.

LIST OF SUPPLEMENTAL TABLES

Supplemental Table 1: Clinical characteristics of the full trial cohort and Abi781 cohort. Treatment arm, age, disease burden, radiotherapy to primary, WHO PS, serum PSA at diagnosis, Gleason score and disease group.

Supplemental Table 2: Overview of 59 signatures included in the study. Signature name, disease the signature was developed in, signature score type and reference for each of the 59 signatures included in this study.

Supplemental Table 3: Wilcoxon rank sum test results comparing continuous signature scores (N=57) by disease state (significance p<0.001). 19 M1 cases where metastatic burden is unknown were excluded from these analyses.

Supplemental Table 4: Wilcoxon rank sum test results comparing continuous signature scores (N=57) by treatment arm (significance p<0.001).

Supplemental Table 5: Results of univariable and multivariable analyses for clinical and pathological adjustment variables included in the Cox models for prognostic outcome and Decipher score in both M0 and M1. Variables included are treatment, age, disease burden (high vs low volume for M1, N1 vs N0 for M0), WHO PS, serum PSA at diagnosis, nsaid use and Gleason score. **A**. Results in M1 disease. **B**. Results in M0 disease. 18 M1 cases where metastatic burden is unknown, three where Gleason score is unknown and one where both are unknown were excluded from these analyses.

Supplemental Table 6: Results of primary and secondary outcome analyses for four primary signatures (AR-A, Decipher, PAM50 and PSC) for Abi781 and Abi723. 18 M1 cases where metastatic burden is unknown, three where Gleason score is unknown and one where both are unknown were excluded from these analyses.

Supplemental Table 7: Results of treatment interaction tests for four primary signatures (AR-A, Decipher, PAM50 and PSC) for Abi781 and Abi723. 18 M1 cases where metastatic burden is unknown, three where Gleason score is unknown and one where both are unknown were excluded from these analyses.

Supplemental Table 8: Results of prognostic testing for primary outcome measures for 55 exploratory signatures in Abi781 and Abi723. Metastasis-free and overall survival are included for M0 and overall survival for M1. 18 M1 cases where metastatic burden is unknown, three where Gleason score is unknown and one where both are unknown were excluded from these analyses.

Supplemental Table 9: Results of treatment interaction tests for overall survival for 55 exploratory signatures in M0 and M1 in Abi781 and Abi723. 18 M1 cases where metastatic burden is unknown, three where Gleason score is unknown and one where both are unknown were excluded from these analyses.

Supplemental Table 10: Results of metastatic state interaction tests for three exploratory signatures prognostic in M0 cohort (IFN_HM, PORTOS and AR_HM). 18 M1 cases where metastatic burden is unknown, three where Gleason score is unknown and one where both are unknown were excluded from these analyses.

Supplemental Table 11: Summary of pathways correlated with hallmark interferon alpha response.

Supplemental Table 12: Comparison of centrally-reviewed Gleason score and locally-reviewed Gleason score as recorded at screening for entry to trial.
